# Compression of Space for Low Visibility Probes

**DOI:** 10.3389/fnsys.2016.00021

**Published:** 2016-03-10

**Authors:** Sabine Born, Hannah M. Krüger, Eckart Zimmermann, Patrick Cavanagh

**Affiliations:** ^1^Centre Attention & Vision, Laboratoire Psychologie de la Perception, Université Paris Descartes, Sorbonne Paris Cité, CNRS UMR 8242Paris, France; ^2^Equipe Cognition Visuelle, Faculté de Psychologie et des Sciences de l’Education, Université de GenèveGenève, Switzerland; ^3^Cognitive Neuroscience, Institute of Neuroscience and Medicine (INM-3), Research Centre JülichJülich, Germany; ^4^Department of Psychological and Brain Sciences, Dartmouth CollegeHanover, NH, USA

**Keywords:** saccades, masking, spatial perception, mislocalization, compression of space, visibility

## Abstract

Stimuli briefly flashed just before a saccade are perceived closer to the saccade target, a phenomenon known as perisaccadic compression of space (Ross et al., 1997). More recently, we have demonstrated that brief probes are attracted towards a visual reference when followed by a mask, even in the absence of saccades (Zimmermann et al., 2014a). Here, we ask whether spatial compression depends on the transient disruptions of the visual input stream caused by either a mask or a saccade. Both of these degrade the probe visibility but we show that low probe visibility alone causes compression in the absence of any disruption. In a first experiment, we varied the regions of the screen covered by a transient mask, including areas where no stimulus was presented and a condition without masking. In all conditions, we adjusted probe contrast to make the probe equally hard to detect. Compression effects were found in all conditions. To obtain compression without a mask, the probe had to be presented at much lower contrasts than with masking. Comparing mislocalizations at different probe detection rates across masking, saccades and low contrast conditions without mask or saccade, Experiment 2 confirmed this observation and showed a strong influence of probe contrast on compression. Finally, in Experiment 3, we found that compression decreased as probe duration increased both for masks and saccades although here we did find some evidence that factors other than simply visibility as we measured it contribute to compression. Our experiments suggest that compression reflects how the visual system localizes weak targets in the context of highly visible stimuli.

## Introduction

Understanding how we perceive and construct the visual space around us is a fundamental task in vision science with a long tradition in philosophy, psychology, neuroscience and other disciplines (see Khurana and Nijhawan, 2010; Melcher, 2011). The visual system has many retinotopically organized representations of the visual field that could encode object locations (Wandell et al., 2007). Despite the explicit location information provided by these spatial maps, objects are sometimes seen at positions other than their true locations. These errors may reveal the mechanisms that the visual system uses to localize objects that have poor position or rapidly changing information. After all, even stimuli that we scarcely see are seen at particular locations—they do not float in our visual experience as ungrounded percepts.

The perceived locations of probes around the time of saccadic eye movements have long been studied for the insight they give us about how spatial coordinates are updated when the retinal image shifts. Indeed, dramatic position errors are reported for a probe stimulus that is briefly flashed around the time of a saccade: the probe is perceived closer to the target of the imminent eye movement than it actually is (e.g., Honda, 1993, 1999; Morrone et al., 1997; Ross et al., 1997; Lappe et al., 2000). In other words, the saccade target seems to attract the flashed probe, a phenomenon known as saccadic compression of space. This is one of the best-known and most-cited effects in the eye movement literature and numerous computational models have been developed to explain it (e.g., Morrone et al., 1997; VanRullen, 2004; Hamker et al., 2008; Richard et al., 2009; Pola, 2011; Cicchini et al., 2013). It is widely assumed that the saccadic compression of space reflects some aspect of spatial updating across eye movements. That is, the transformation from pre- to post-saccadic coordinates guided by extraretinal oculomotor signals (e.g., eye position or efference copy signals) that interact with the visual input.

Recently, we have reported a mask-induced compression effect in a condition without saccadic eye movements (Zimmermann et al., 2013, 2014a; Born et al., 2015). We first presented a visual reference stimulus in the periphery, followed by a flashed probe and a random-texture mask. Participants held fixation throughout each trial. Although the reference was irrelevant to the task, participants’ localization responses were strongly biased towards the reference. Our findings question the widely held assumption that the perceptual compression of space is a phenomenon closely linked to movement-dependent extraretinal signals like corollary discharge or changes in eye position. Rather, compression of space (saccade or mask-induced) could be the signature of a much more general mechanism.

To reveal the specifics of this mechanism, one may first ask if there are any characteristics common to both saccades and masks that are critical for compression to occur. On the one hand, saccades and masks both disrupt the visual input stream. Thus, compression could be the signature of a correspondence matching mechanism that maintains object identities across visual discontinuities (Ullman, 1979; Zimmermann et al., 2014a). Alternatively, it is well known that both masks and saccades reduce the visibility of simultaneously presented stimuli (e.g., Diamond et al., 2000; Breitmeyer and Öğmen, 2006). Accordingly, it may simply be the detrimental effect of masks or saccades on the visibility of the probe that induces compression where a localizing mechanism for the weak stimulus is biased by a nearby strong reference.

Here, we examined the role of transient discontinuities and probe visibility in producing compression. Experiment 1 suggests that transient visual disruptions are not necessary. Experiment 2 confirms this observation and demonstrates similar modulations of compression across different levels of probe visibility in saccade and masking conditions as well as a condition where only probe contrast varies in the absence of saccades and masks. Experiment 3 demonstrates weaker compression with longer probe durations and a similar time course of compression as a function of probe duration and timing for both saccades and masks. It also gives some evidence that compression may not be solely driven by the visibility of the probe. The experiments provide further evidence for a common mechanism underlying saccadic and other forms of compression of visual space.

## Experiment 1: Effect of Visual Transients

In Experiment 1, we examined whether transient disruptions of the visual input stream (e.g., masks) are critical for compression to occur. In previous experiments, we used a full-screen mask. Here, we varied the regions of the screen covered by the mask. Specifically, we tested: (a) whether mask and probe have to spatially overlap, that is, whether the mask has to interfere directly with the visual signal of the probe; (b) whether a transient visual disruption around fixation (i.e., the current focus of the visual input stream) or any part of the visual field (e.g., the upper and lower third of the screen) can induce compression; and (c) whether a mask was necessary at all. To control for effects of probe visibility, we manipulated the probe’s contrast using a staircase procedure to maintain an approximately constant level of visibility individually for each participant across trials in each condition.

### Methods

#### Participants

Eight participants were tested in Experiment 1 (four women, one author, aged between 25 and 36 years). All reported normal or corrected-to-normal vision. For all experiments reported in this study, observers gave written informed consent prior to participating and the procedures followed the principles laid down in the Code of Ethics of the World Medical Association (Declaration of Helsinki).

#### Apparatus

Subjects were seated 57 cm from a 22″ CRT monitor with head stabilized by a chin- and headrest. Stimuli were presented with a monitor refresh rate of 120 Hz at a resolution of 1024 × 768 pixels. The experiment was programmed in Matlab (The MathWorks Inc., Natick, MA, USA) using the Psychophysics and Eyelink Toolbox extensions (Brainard, 1997; Pelli, 1997; Cornelissen et al., 2002). Eye movements were recorded using an EyeLink1000 desk-mounted eye tracker (SR-Research Ltd., Mississauga, ON, Canada) at a sampling rate of 1000 Hz.

#### Stimuli, Design and Procedure

**Figure 1:**
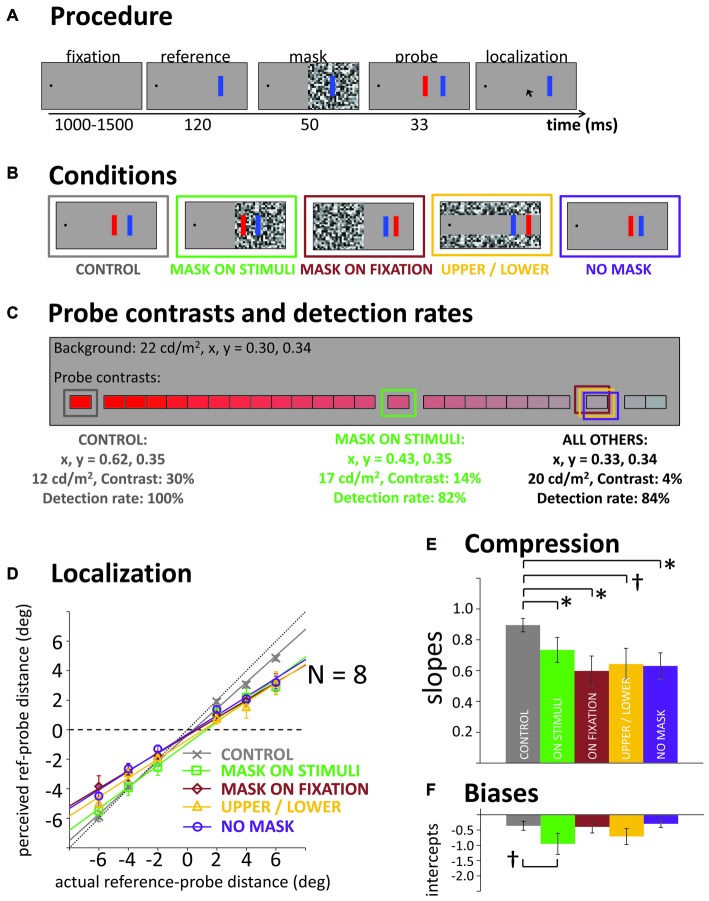
**(A)** General procedure in all experiments. Participants indicate at the end of a trial where they had seen the flashed red probe bar. **(B)** Illustration of the five masking conditions of Experiment 1. **(C)** Illustration of the color/luminance contrast scale used to adjust probe visibility in Experiments 1 and 2. Framed in color: average contrast levels towards which the staircases converged in the different conditions of Experiment 1, their characteristics (CIE1931 color coordinates, luminance, Michelson contrast to background) and actual probe detection rate. **(D)** Actual vs. average reported probe distance from the reference (negative values: probe closer to fixation than reference) in the five conditions and corresponding linear fits. Horizontal black dashed line illustrates a slope of *b* = 0 (i.e., full compression), black dotted diagonal line illustrates a slope of *b* = 1 (i.e., no compression). **(E,F)** Slopes and intercepts of the linear regression lines, averaged across the individual fits for our eight participants. All error bars: standard error of the mean. *p < 0.05, ^†^*p* < 0.10.

The general procedure is illustrated in Figure 1A. A trial started with the presentation of a black fixation square (0.5° side length), 10° to the left or right of the screen center (counterbalanced across participants). After a variable delay between 1000 and 1500 ms, a blue vertical reference bar (side length: 0.6 × 3°) was presented at a horizontal distance of 15° from fixation (i.e., 5° from screen center, opposite to fixation). Participants were instructed to fixate the black square (controlled by eye tracking) and were asked to ignore the onset of the reference. Then, 120 ms after reference onset, a mask (gray squares of randomized luminance, 0.7° side length) was presented for 50 ms, followed by a red probe bar of the same size as the reference, briefly flashed for 33 ms. In separate blocks, we tested masks covering different areas of the screen (see Figure 1B): the mask was either presented on the same screen hemifield as the reference and probe (mask on stimuli), on the fixation hemifield, or the mask consisted of two horizontal bands, covering the upper and lower thirds of the screen, but not overlapping with any stimulus. Additionally, two blocks without any mask were run: one block with a low contrast probe (adjusted by staircase; see below), and one block in which the probe was always presented at high contrast, serving as control condition. In those blocks, instead of the mask, the reference stayed on screen for an additional 50 ms without any further event before the probe was presented. Thus, in all conditions the probe appeared 170 ms after the reference.

The probe’s location was pseudo-randomly chosen on each trial from six possible horizontal offsets: it was presented at a distance of either −6, −4, −2, 2, 4, or 6° horizontally from the reference bar (negative values: more foveal, positive values: more peripheral than the reference). After the probe, the mouse cursor appeared. Participants were required to indicate where they had seen the probe with a click on the corresponding location on the screen. For each trial, the cursor was initially placed at a random position, maximum distance ±4° horizontally from the reference. Additionally, in line with previous experiments (Born et al., 2015), we presented a response grid consisting of horizontal and vertical dotted lines, covering the entire screen (lines 1.9° apart, with one of the horizontal lines on the horizontal meridian, and one of the vertical lines passing through the reference). With onset of the response display, participants were free to move their eyes.

Importantly, if participants had not seen the probe on a given trial, they were instructed to click on the fixation square. Accordingly, probe contrast was adjusted individually for each participant and block (except in the high contrast control). Our aim was to roughly equate visibility across conditions. With each click on the fixation square indicating an unseen probe, color contrast was increased on the next trial. Contrast was reduced when the participant gave four localization responses in a row (i.e., staircase procedure following a “4-down, 1-up” rule, targeting the 84% detection threshold for the probe). We used 25 discrete contrast levels that at the highest level was a dark saturated red, at Michelson contrast of 30% with the lighter background, then approached the background in luminance and color so that the last step was approximately 1/25th of that difference in contrast (1.2%) and saturation from the background (see Figure 1C).

Participants completed one block in each of the five conditions (order counterbalanced across participants). Within a block, each of the six probe locations was tested 20 times, resulting in a minimum of 120 trials per block. Trials in which participants clicked on fixation (“not seen”) and trials in which the eye tracker detected a violation of the fixation instruction were repeated at the end of a block.

### Results and Discussion

#### Error Trials and Probe Detection

Due to blinks or breaks of fixation, 1.6% of trials were excluded from analysis. Also, the first 20 trials in each block were discarded to allow the staircase procedure to approach the contrast level producing the targeted 84% probe detection rate. In the control block, the probes presented at maximum contrast (30% luminance contrast, fully saturated red) were detected in 100% of cases (see Figure 1C). The staircases in the three masking conditions in which the mask did not overlap with the probe all converged towards the same average contrast (4% contrast, see Figure 1C) and successfully produced the targeted 84% detected probes. In the mask-on-stimuli condition, the probe detection rate was slightly lower (82%) and the probe had to be presented at a much higher contrast (14%).

#### Localization and Compression

Figure 1D illustrates actual against mean perceived probe locations averaged across our eight participants. Fitting linear regressions to the data, the slope of the regression lines can be taken as an estimate of compression: if perception was veridical and without compression (i.e., actual = perceived location), the slope should be close to *b* = 1, if all stimuli were perceived fully compressed towards one single location, a flat line should emerge with a slope close to *b* = 0. To test for differences in compression across conditions statistically, we performed individual fits to each participant’s data. Figure 1E shows the average slopes in the respective conditions. The high contrast control condition showed a steeper slope, that is, less compression as all of the other conditions with reduced probe detection rate (see Figure 1E). A repeated-measures one-way analyses of variance (ANOVA) across all conditions, *F*_(4,28)_ = 2.73, *p* = 0.047, and *post hoc* pairwise *t*-tests confirmed the steeper slope in the control (all *t*s_(7)_ > 2.42, *p*s < 0.046, except control vs. upper/lower: *t*_(7)_ = 1.91, *p* = 0.097). However, when omitting the control condition from the ANOVA, the significant main effect of condition disappeared, *F*_(3,21)_ = 0.68, *p* = 0.573. Thus, it did not matter if the mask was overlapping with the probe, overlapping with fixation or if there was a mask at all: there was significant compression in all conditions. In other words, there was no evidence that a transient disruption of the visual input stream by the mask was critical for compression to occur.

Figure 1D also illustrates that for some conditions, compression was seen more in the mislocalizations of the more peripheral probes than the more foveal ones (see, e.g., the “mask-on-stimuli” condition). Probes that were presented more foveally than the reference were localized with less error or sometimes no error at all and the asymmetry in mislocalizations leads to overall negative intercepts in the fitted regression lines (see Figure 1F). This negative intercept and the smaller deviation for more foveal probes may be indicative of foveal biases where all brief flashes are seen closer to the fovea than they actually are (Mateeff and Gourevich, 1983; van der Heijden et al., 1999; Kerzel, 2002; Born et al., 2015). Alternatively, it may be due to lower probe visibility for the more peripheral than the more foveal probes (this was not taken into account in the staircase visibility procedure). In Experiment 2, we therefore used separate staircases for more foveal vs. more peripheral probes.

## Experiment 2: Effect of Probe Visibility

In Experiment 2, we extend our finding of compression without transients and directly examined the role of visibility. We pitted saccadic against mask-induced compression and mislocalizations without visual disruptions at different levels of visibility.

### Methods

Seven participants completed Experiment 2 (all women, aged between 25 and 35 years, two authors, three had already participated in Experiment 1). All reported normal or corrected-to-normal vision. Apparatus, stimulus characteristics and procedure were the same as in Experiment 1 with the following exceptions. We ran mask (full screen), saccade and “none” (no mask, no saccade, no other transient) blocks, varying the visibility of the probe. In the saccade condition, participants were asked to make an eye movement towards the blue reference bar as soon as it appeared. The probe was always shown when the saccade was detected online. Because of the unavoidable delay between saccade onset, its online detection and waiting for the next monitor refresh, the probe appeared on screen on average 25 ms after saccade onset, and disappeared around 12 ms before saccade offset (mean saccade duration: 70 ms). On 7% of trials, the probe was still on screen when the eyes landed (those trials were not excluded as saccadic compression can typically still be found for some time after saccade offset). For some conditions in Experiment 1, the deviations from veridical locations were larger for the more peripheral than the more foveal probes. This may have been due to lower probe visibility for the more peripheral than the more foveal probes because of the larger eccentricity from fixation. To better equate visibility across eccentricity in this second experiment, we always ran two interleaved staircases in a block instead of only one: one for stimuli more foveal and one for stimuli more peripheral than the reference. For each condition (mask, saccade, none), we ran two separate blocks in which the staircases followed different rules: a “5-down, 1-up” rule or a “2-down, 1-up” rule. Thus, we ensured that a large range of probe contrasts was presented, low as well as medium contrast levels, but still individually adjusted to each participant’s overall probe detection performance separately for inward (closer to the fovea than the target or the reference) and outward probes. As staircases are inherently noisy, contrast ranges in the different staircase blocks often overlapped widely, though. Thus, to clearly distinguish low from medium contrast trials, we performed *post hoc* median splits for each participant. In total, the experiment consisted of seven blocks (one control, two saccade, two mask, two “none”). Trials with “unseen” responses or violations of the fixation instruction were repeated at the end of a block. In the saccade condition, trials were also repeated when a violation of the eye movement instruction was registered: anticipatory saccades (latency < 100 ms), breaks of fixation before reference onset, blinks, or no saccade within 600 ms after reference onset.

### Results

#### Error Trials and Probe Visibility

**Figure 2:**
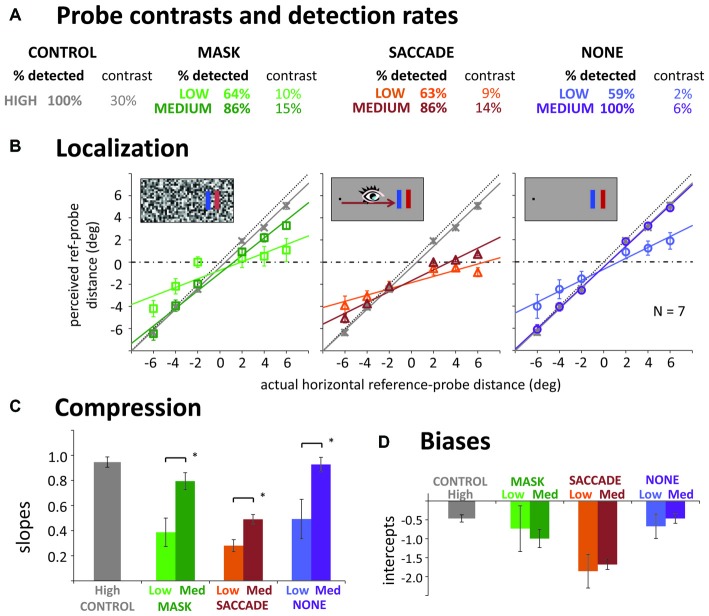
**Results of Experiment 2. (A)** Detection rates and mean contrast levels for the probes in low and medium contrast conditions. Contrast was varied along a combined luminance and color contrast scale (see Figure 1); for simplicity, only luminance (Michelson) contrast is reported here. **(B)** Actual vs. average reported reference-probe distances (mask, saccade, none) and the corresponding linear fits, separate for low contrast (pale symbols) and medium contrast trials (dark symbols). Gray data in each panel illustrate the high contrast control. **(C,D)** Slopes and intercepts of the linear regression lines. All error bars: standard error of the mean. *p < 0.05.

Trials immediately discarded and repeated due to violations of the fixation or saccade instructions amounted to 3.5% in the control, 3.7% in the mask, 7.1% in the saccade, and 2.0% in the “none” condition. As in Experiment 1, participants almost never missed the probe in the high contrast control condition (see Figure 2A). After median splits into low and medium contrast trials (individually for each participant; separate for the three conditions and for more foveal vs. more peripheral probes), similar probe detection performance around 60–65% was obtained in the mask, saccade and “none” conditions on low contrast trials. On medium contrast trials, performance was around 86% in the mask and saccade conditions. However, in the “none” condition, detection performance was close to perfect. Not surprisingly, average contrast levels leading to these detection rates were lower in the low than in the medium contrast condition. They were also lower in the “none” condition compared to saccade and mask conditions. Interestingly, contrasts did not differ substantially in the mask and saccade conditions, suggesting that masks and saccades diminished perceived probe contrast to a similar degree.

#### Localization and Compression

Figure 2B illustrates actual against mean perceived probe locations and the corresponding linear fits. Slopes of these fits are summarized in Figure 2C. To first evaluate whether we had compression effects in the different conditions, we compared the control slope to the six other conditions. For low contrasts, all slopes were significantly shallower than the control slope, suggesting compression in all three manipulations (pairwise *t*-tests: all *p*s < 0.040). For medium contrasts, only the saccade condition had a significantly shallower slope than control, *t*_(6)_ = 11.24, *p* < 0.001. The difference from control in the mask condition was only marginally significant, *t*_(6)_ = 2.00, *p* = 0.099, and there was no difference in the “none” condition, *t*_(6)_ = 0.35, *p* = 0.741. To compare the effects of our contrast manipulation across mask, saccade and the “none” condition, we next performed a 3 (condition) × 2 (contrast) repeated measures ANOVA: we obtained a significant main effect of contrast, *F*_(1,6)_ = 35.45, *p* = 0.001, indicating stronger compression with lower probe contrasts. The interaction was not significant, *F*_(2,12)_ = 1.09, *p* = 0.369, suggesting that a contrast effect was obtained in all three conditions (confirmed by paired *t*-tests, all *p*s < 0.022), and roughly of similar magnitude. The main effect of condition was also significant, *F*_(2,12)_ = 9.57, *p* = 0.003. *Post hoc* *t*-tests, revealed that the effect was due to shallower slopes in the saccade (*p*s < 0.012), than the mask and “none” conditions, which did not differ significantly from each other (*p* = 0.165). In sum, we could confirm our finding of a compression effect without any transient disruption of vision. Further, we found compression to be strongly modulated by probe visibility for saccades, masks and in the “none” condition.

Even though we equated visibility for the more foveal and more peripheral probes by employing separate staircase procedures, we still find large negative biases (intercepts; see Figures 2B,D), especially in the saccade condition. These negative shifts are compatible with a foveal bias (Mateeff and Gourevich, 1983; van der Heijden et al., 1999; Kerzel, 2002; Born et al., 2015) that affects all the probes, moving a fairly linear, compressed pattern of localization (slope less than 1) away from crossing through the reference position to values that are all 0.5–1.0° closer to the fovea. Experiment 3 will further look into this issue but we can at least rule out the possibility that this bias was caused by less visible probes in the periphery.

## Experiment 3: Time Course of Probe Visibility Effects

Experiment 3 elaborates on the time course of compression effects across different probe visibility levels by varying the interval between probe presentation and the onset of the mask or saccade. To corroborate that our findings in Experiments 1 and 2 are effects of the general visibility of the probe and not specific to contrast manipulations, here we took a different approach and manipulated probe visibility by varying probe duration. Typically, saccadic compression studies use even shorter probe durations (<20 ms) than the 33 ms we used in Experiments 1 and 2. To our knowledge, effects of probe duration have not been tested systematically, yet.

### Methods

Five participants completed Experiment 3 (all women, aged between 20 and 34 years, including the same two authors already tested in Experiment 2). All reported normal or corrected-to-normal vision. Apparatus, stimulus characteristics and procedure were the same as in the previous experiments with the following exceptions. Two of the five participants were tested on a different CRT monitor (21" NEC MultiSync FE2111SB; resolution of 1280 × 1024 pixels) running at 85 Hz instead of 120 Hz. The reference bar was yellow and the probe bar was blue. The probe could be presented at four instead of six possible offsets: either −5, −2, 2, or 5° from the reference. We ran a saccade, a mask and a control condition where the probes were always presented at the same contrast (5 cd/m^2^, *x* = 0.14, 0.08) with respect to the background, but with varying duration (randomly intermixed): a short duration of about 20 ms (17/24 ms, depending on exact screen refresh rate), a medium duration of about 45 ms (42/47 ms) and a long duration of about 100 ms (94/100 ms). Also, the probe was not presented at a fixed point in time, but at one of five possible delays of 25, 100, 175, 250 or 325 ms after the reference. In the masking condition, the mask was always presented at a fixed delay of 175 ms after reference onset. As before, participants could indicate not to have seen the probe by clicking on the fixation square, albeit, without any resulting adjustments of probe characteristics. To establish a full time course, participants were subject to about 6 h of testing (split up into sessions lasting 60–90 min), amounting to 3000–3600 trials per participant (approximately half of the trials in the saccade condition, and one fourth in control and mask conditions, respectively).

### Results

#### Error Trials and Probe Visibility

Trials immediately discarded and repeated due to violations of the fixation or saccade instructions amounted to 12.6% in the control, 8.4% in the saccade, and 5.3% in the mask condition.

#### Localization, Visibility and Compression

**Figure 3:**
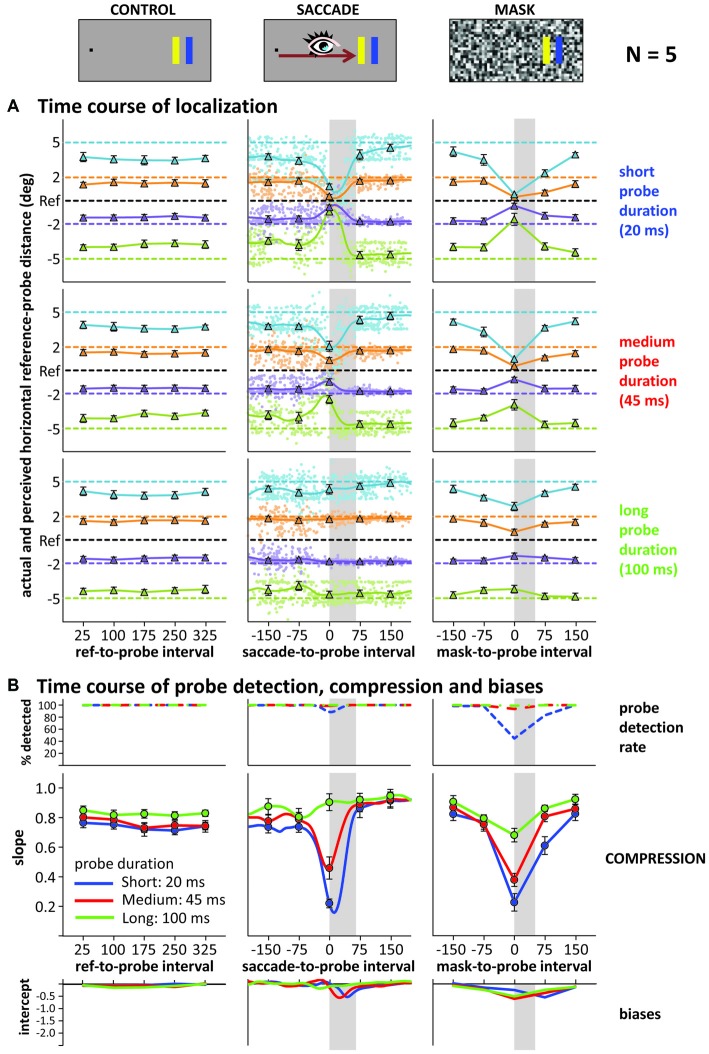
**Results of Experiment 3. (A)** Actual (horizontal dashed lines) vs. average reported (triangles and solid lines) reference-probe distance (control, saccade, mask) across time: reference-, saccade- or mask-to-probe interval (negative values: probe presented before saccade or mask; gray shaded areas illustrate the average saccade and mask duration). Depicted are data for short (upper), medium (middle) and long (lower) probe durations. Data is averaged across all five participants. Saccade graphs: small dots represent individual trial data, smooth curves the weighted running average. **(B)** Detection rates, compression indices (slopes) and biases (intercepts) across time and probe duration in control (left), saccade (middle; smooth curves: running averages, circles: time points as in control and mask conditions) and mask (right) condition. All error bars: standard error of the mean across participants.

Figure 3A illustrates the time course of localization responses in the control, saccade and mask conditions (left, middle, right columns), separate for short medium and long probe durations (upper, middle, lower panels), averaged across all five participants. Data in the saccade and mask conditions are re-aligned to saccade and mask onset: negative intervals denote that the probe was presented before saccade/mask onset. As this realignment produces large trial-by-trial variations in the saccade-to-probe interval (pale dots in the graphs: responses on individual trials), we calculated a weighted running average in the saccade condition using a moving Gaussian window with a standard deviation of 20 ms. The time points along this smooth curve that were also tested in the control and mask conditions are highlighted by the triangles. Horizontal dashed lines in each panel illustrate the actual locations of reference and probes. The graphs show that in the control condition, localization responses were largely independent of probe duration and the delay between reference and probe. For saccade and mask conditions, similar localization responses as in the control condition were observed when the probe was presented either more than 75 ms before or after the saccade/mask. However, probes presented close in time to the onset of saccades or masks were mislocalized. The convergence of localization responses around the reference location was very strong for short duration probes in both saccade and mask conditions and slightly less pronounced for medium duration probes. For long probe durations, compression seems absent in the saccade condition, but still slightly present in the mask condition.

**Figure 4:**
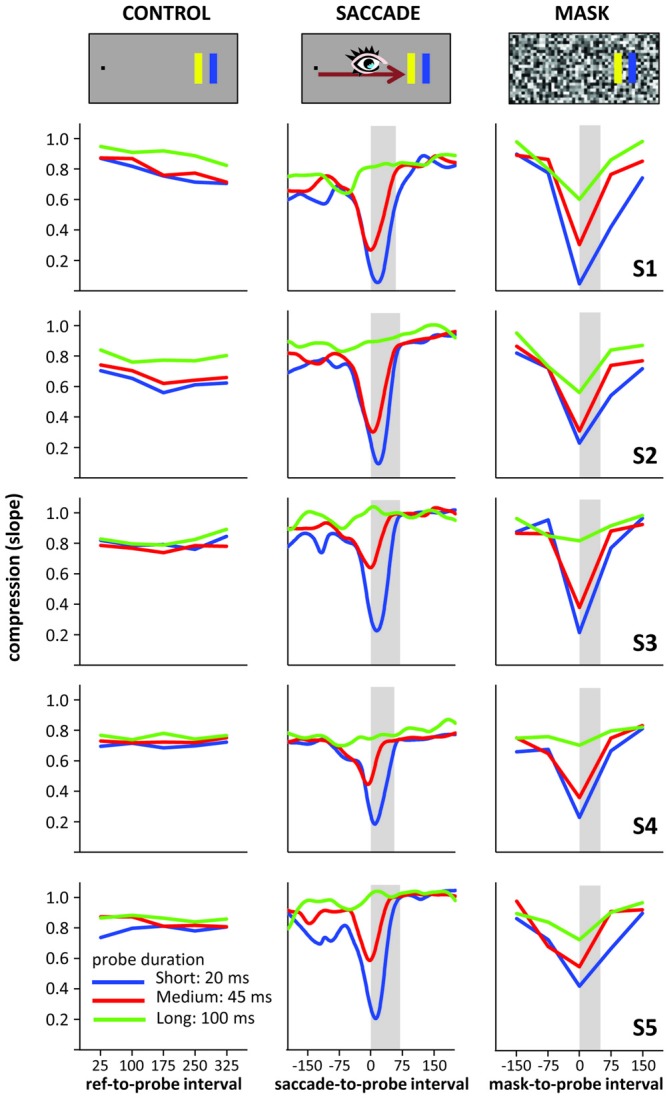
**Individual compression data from the five subjects (S1–S5 top to bottom) of Experiment 3**.

To better characterize compression across time, we again summed up across the different probe locations by fitting linear regressions (actual vs. perceived location) to the data at each time point (including “running” regressions based on the running averages), taking the slope of these regressions as compression index. Figure 3B depicts the time course of these indices: the larger the “dip” in the graphs, the stronger the compression effect. In the control condition, no dip occurred, whereas the saccade and mask conditions show large dips. These dips all reach their maximum roughly around saccade or mask onset and their depth scales with probe duration: the shorter the probe presentation, the stronger the compression effect. Of note is that the compression for the 20 ms and 45 ms probe durations differed when presented at saccade/mask onset, even though both fully fell into the masking period (50 ms) or within the duration of the saccade (~65 ms). Figure 4 illustrates that the time course and compression patterns of individual subjects closely resemble the averaged data. Although similar, there are also some differences in the saccade and mask conditions. For instance, mask-induced compression seems to last slightly longer than saccadic compression: 75 ms after saccade onset, slopes are again close to one whereas there is still some compression in the masking conditions 75 ms after mask onset, at least for short duration probes. Also, as already mentioned, there is some compression in the masking condition even for the long 100 ms duration probes, but none in the saccade condition. With probes presented at saccade/mask onset and 75 ms after saccade/mask onset giving the most interesting patterns, we first conducted a 2 (condition: saccade vs. mask) × 3 (duration: short, medium, long) × 2 (time point: onset vs. onset +75 ms) repeated-measures ANOVA on the slopes that confirmed a significant three-way interaction, *F*_(2,8)_ = 12.77, *p* = 0.003. At saccade/mask onset, separate one-way ANOVAs revealed highly significant effects of duration in both the saccade and mask conditions, *F*s_(2,8)_ > 51.38, *p*s < 0.001. At 75 ms after saccade/mask onset, the main effect of duration remained significant in the mask condition, *F*_(2,8)_ = 17.98, *p* = 0.001, but dropped to a marginally significant level in the saccade condition, *F*_(2,8)_ = 3.64, *p* = 0.075. Further, comparing saccade and mask effects directly, mask-induced compression was found to be stronger at saccade/mask onset for the long duration probes, *t*_(4)_ = 4.42, *p* = 0.012, and 75 ms after saccade/mask onset for the short duration probes, *t*_(4)_ = 5.04, *p* = 0.007. No further pairwise comparison reached significance.

Although we did not adjust probe characteristics based on participants’ probe detection rates in Experiment 3, we nevertheless gave participants the opportunity to report whether they saw the probe with a click on fixation (see Figure 3B). Only the short duration probes in the masking condition produced a substantial drop in probe detection rate when presented close in time to mask onset. Interestingly, for both masks and saccades, medium duration probes that were detected almost 100% of the time still produced compression effects when presented around saccade/mask onset.

**Figure 5:**
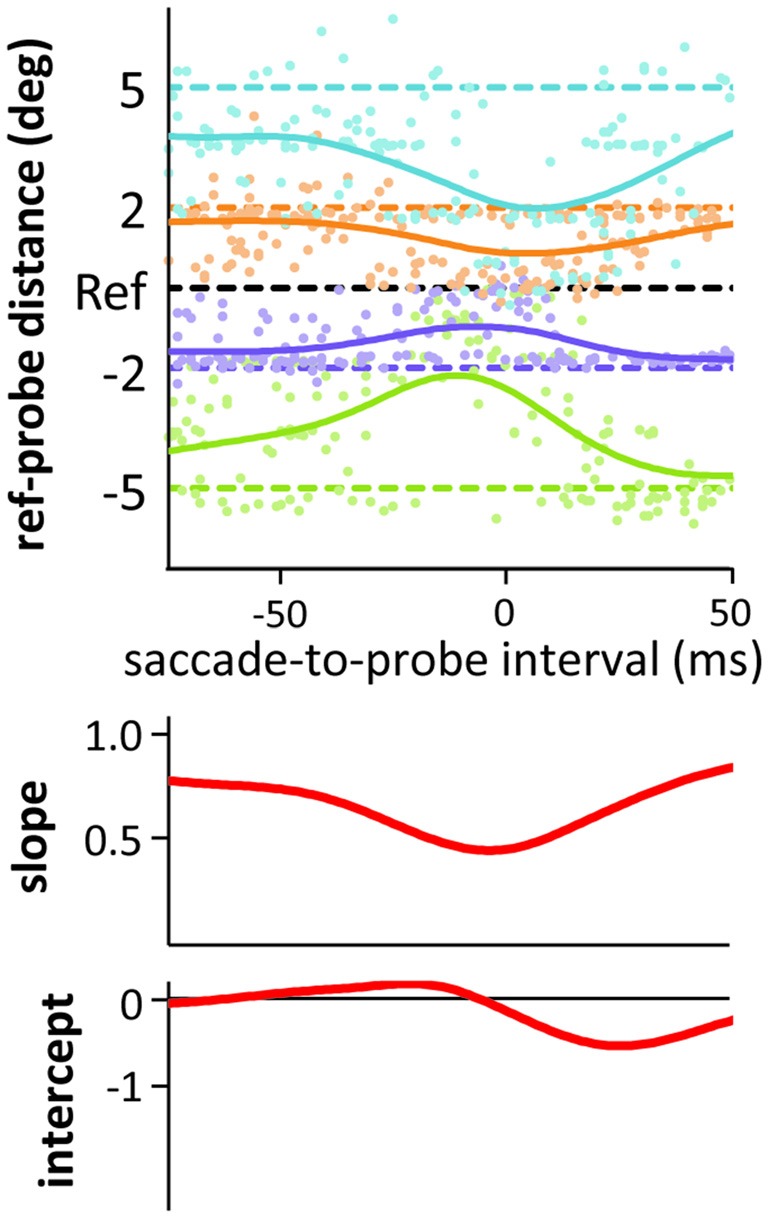
**Results from the medium duration probes in the saccade condition of Experiment 3.** Only the saccade-to-probe intervals from −75 to 50 ms with respect to saccade onset are shown. All conventions as in Figure 3.

Figure 3B also shows the intercepts of our linear fits across time (including a “running” intercept in the saccade condition), that is, a measure of uni-directional bias or asymmetry (see Experiments 1 and 2) in the localization responses. During the saccade and at mask onset or slightly after, negative intercepts emerged. A closer look at the time course of localization and the resulting compression indices (see Figure 5 for an example showing the data of the medium duration probe in the saccade condition, but zooming in on the time around saccade onset) reveals that this effect is partly due to mislocalizations for the more peripheral probes starting slightly later and lasting slightly longer than for the more foveal probes (compare green and blue curves in Figure 5). Although not widely discussed in the literature, this pattern can be found in the data of other compression studies as well (see e.g., Lappe et al., 2000; Figure 1; or Maij et al., 2011; Figure 2). The asymmetry and resulting negative intercepts are in line with the results from Experiments 1 and 2 where we presented the probe rather late: during the saccade or at mask offset (although the asymmetry was never as strong as the one we observed in the saccade condition in Experiment 2).

**Figure 6:**
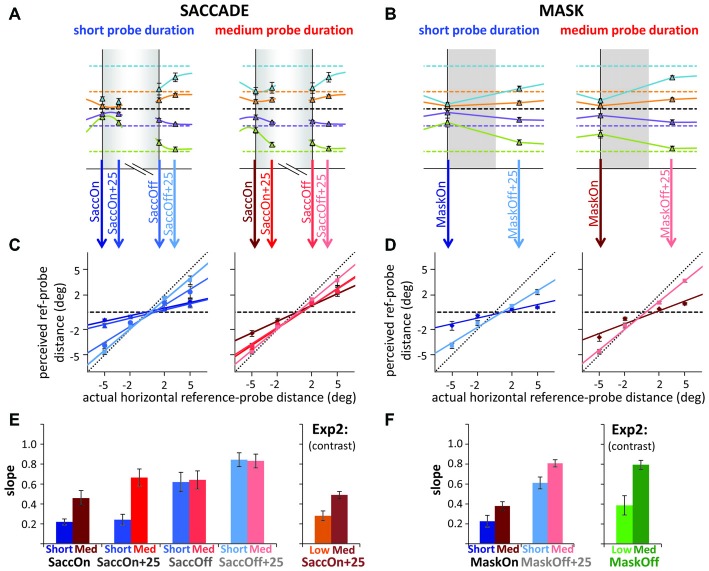
**Fine temporal scale analysis around saccade/mask onset and offset for short and medium probe durations in Experiment 3. (A,B)** Actual (horizontal dashed lines) vs. average reported (triangles and solid lines) reference-to-probe distance (**A**: saccade, **B**: mask) at saccade/mask onset, 25 ms after saccade onset, at saccade offset and 25 ms after saccade/mask offset (gray shaded areas illustrate saccade/mask duration; data in the saccade condition is time-locked either to saccade onset as in previous figures; or recoded, time-locked to saccade offset on a trial-by-trial basis such that the curves do not smoothly connect to each other, also illustrated by the white gap in the gray shade in **A**). **(C,D)** Actual vs. average reported reference-to-probe distance and corresponding regression lines fitted to the data. Left graphs (blue): fits for the short duration probes, right graphs (red): fits for medium duration probes. The different time points are marked with different shades of blue/red. **(E,F)** Slopes of the regression lines from **(C,D)** along with slopes from Experiment 2 for low and medium contrast probes. All error bars: standard errors of the mean.

Finally, in Figure 6 we replotted the data around saccade/mask onset and offset to compare specific time points for short and medium duration probes to the results from the low and medium contrast probes of Experiment 2. As probes in Experiment 2 were presented during the saccade or at mask offset, we recoded part of the saccade data of Experiment 3, time-locking it on a trial-by-trial basis to saccade offset. Figures 6A–D illustrates the steps to arrive at the compression indices (slopes) depicted in Figures 6E,F. In the saccade condition of Experiment 2, probes were presented around 25 ms after saccade onset. Figure 6E shows that compression indices from Experiment 2 roughly match those found in Experiment 3, resembling most the results observed at saccade onset. Interestingly, compression and the differences between duration conditions were already greatly reduced at saccade offset in Experiment 3. In contrast, in the mask conditions of Experiment 2, the probes were presented simultaneously with mask offset. Again, Figure 6F shows that compression indices for the mask conditions of Experiment 2 roughly match those in Experiment 3, falling in between those observed at mask onset and 25 ms after mask offset. Overall, Figures 6E,F suggest that the stronger compression found for saccades compared to masks in Experiment 2 could indeed be partly due to the slightly earlier presentation of probes in the saccade conditions. In Experiment 2, we observe significant compression in the mask conditions with the probe at mask offset only because mask-induced compression slightly outlasts saccadic compression.

## General Discussion

In three experiments, we addressed the role of transient disruptions of the visual input stream and the importance of probe visibility on spatial compression, where spatial compression is the systematic mislocalization of briefly flashed probes toward visual references typically observed in the context of saccades (Morrone et al., 1997; Lappe et al., 2000) and masks (Zimmermann et al., 2013, 2014a; Born et al., 2015). In Experiment 1, we found compression even without a visual disruption from either a saccade or a mask. It was sufficient to present the probe at low contrast, reducing its detection rate, to obtain compression-like mislocalizations. Experiment 2 confirmed compression for low contrast probes without saccade or mask and demonstrated modulations of compression with probe contrast in all three cases (mask, saccade, no disruption): stronger compression was observed with lower detection rates. Finally, we manipulated probe duration in Experiment 3 and looked at compression effects in saccade and mask conditions across time. Compression increased with shorter probe durations just as it had with lower contrast. Compression peaked at saccade/mask onset in all conditions and showed a roughly similar time course in the context of saccades and masks. Interestingly, some conditions (with probes very close to the saccade or mask onset) still produced compression in Experiment 3 even with perfect detection rates of the probe. This suggests that saccades and masks have an effect on the localization of the probe that is not completely accounted for by their effects on the probe’s detectability.

### The Role of the Visual Reference: Biasing Perceived Location

In the current experiments, the reference bar acts as a strong visual attractor. For saccadic compression, it has been suggested that attraction is dependent on extra-retinal signals: corollary discharge or predictive eye position information that build up strong modulatory signals around the endpoint of the saccade (e.g., Morrone et al., 1997; Lappe et al., 2010). Accordingly, compression was found to be centered on locations in empty space in saccadic adaptation and anti-saccade paradigms (Awater and Lappe, 2004; Awater et al., 2005). In contrast, however, Zimmermann et al. (2014b) have recently shown that saccadic compression is greatly diminished when no visual reference is presented in the visual field, but saccades are directed towards the empty screen center. Compression re-emerged when irrelevant visual reference stimuli were presented, albeit with attraction towards the reference, not the saccade endpoint at the center of the screen (see also Cicchini et al., 2013; Luo et al., 2014). These findings suggest that even in saccadic compression, strong visual signals may override weaker extra-retinally generated signals to act as attractors (see also Lappe and Hamker, 2015, this issue). Our effects without saccades show that strictly movement-related extra-retinal information (eye position or corollary discharge) is not even necessary to observe compression-like mislocalizations.

Importantly, we do not rule out the involvement of oculomotor structures or other types of extraretinal signals in compression. To the contrary, it is very likely that the onset of our reference generates a sharp neural response in saccade or priority maps in oculomotor structures like the superior colliculi or frontal eye fields (e.g., Sparks, 2002; White and Munoz, 2011; Schall, 2014). However, there is also now a broad consensus that oculomotor structures like SC or FEF are not only vital for saccade programming, but also play a crucial role in covert attentional selection and visual processing (e.g., Corbetta et al., 1998; Awh et al., 2006; Fecteau and Munoz, 2006; Krauzlis et al., 2013; Schall, 2014). Our effects are therefore not saccade-specific, at least not to the same extent as eye position or corollary discharge signals which should be dependent on the actual execution of eye movements. In contrast to more general (covert or overt) selection mechanisms, those eye position or corollary discharge signals are thought to mediate spatial updating across eye movements (e.g., Matin et al., 1970; Dassonville et al., 1992; Honda, 1993; Morrone et al., 1997; Hamker et al., 2008; Teichert et al., 2010; Pola, 2011; Ziesche and Hamker, 2011). Given the retinotopic organization of many cortical areas, spatial updating of retinotopic coordinates across eye movements is clearly required. However, we suggest that compression effects (although certainly being very striking phenomena and interesting to study in their own right) may not be related to these updating processes, as they also occur in situations in which no spatial updating is needed such as mask-induced compression or motion-induced compression (see also Ostendorf et al., 2006; Shim and Cavanagh, 2006) and they are greatly reduced when spatial updating is needed but no visual reference is available (Luo et al., 2014; Zimmermann et al., 2014b).

### The Role of Probe Visibility: Diminishing the Quality of the Probe’s Spatial Representation

In addition to a strong attractor signal, it seems that a weak representation of the probe makes it susceptible to mislocalizations. Previous research has shown that saccadic compression decreases with increasing probe contrast and luminance (Michels and Lappe, 2004; Georg et al., 2008). Here, we complement this earlier work by showing gradual modulations of compression with probe visibility (contrast and duration), not only in the context of saccades but also masks. Low contrast probes caused compression effects even without saccades or masks (Experiments 1 and 2). It seems plausible to assume that reducing contrast or duration leads to an overall impoverished probe signal, affecting many aspects of its perceptual representation simultaneously. Consequently, determining its color, size, shape—but importantly also its location—becomes more difficult, up to the point where the probe’s presence cannot be detected anymore.

In Experiment 3, saccades as well as masks produced compression even when the detectability of the probe was close to perfect, suggesting an effect of masks and saccades that goes beyond a mere reduction in visibility. The observation contrasts with our findings in Experiment 2 where there was no compression in the mask condition when probe detectability was around 85% while saccades still produced compression at a similar probe detection rate. The time course of compression found in Experiment 3 may partly explain the conflicting results: given that the probe was always presented at mask offset (but still during the saccade in the saccade condition) in Experiment 2, the lower compression strength may have reduced the contributions of any additional factors compared to measurements at mask onset.

How can saccades or masks cause compression with perfectly detectable probes? We tried to equate visibility in our experiments by equating detection rates. This might be a rather coarse approximation, though, as visibility goes beyond mere detectability: two stimuli that are equally easy to detect may still differ in visibility (e.g., in terms of perceived color saturation or sharpness around the edges). Along these lines, the onset of a saccade or mask may diminish the quality of the probe’s spatial representation more than, for instance, its color or shape information. Several factors can lead to a reduction of probe visibility at the time of saccades: saccadic suppression mediated by extraretinal signals (e.g., Diamond et al., 2000), backward masking through the post-saccadic retinal image (Matin et al., 1972) and the high-speed motion of the eye producing retinal smear, resulting in a blurred perceived image. But it has also been observed that not all attributes are equally affected: perceptual sensitivity for low spatial frequencies and transients is reduced more strongly than, for example, color sensitivity (Burr et al., 1994). Masks also affect some stimulus attributes more than others (Breitmeyer and Ganz, 1976; Enns and Di Lollo, 2000). Based on these less affected attributes, the probe’s presence may have been reliably detected in most conditions of Experiment 3. But a poor spatial or transient onset signal could have made it more susceptible to influences from the reference. Simple reductions in stimulus contrast or duration may not have the same effect until the stimulus approaches detection threshold.

It has been suggested that compression reflects an underestimation of relative distance rather than a mislocalization of the absolute position of the probe (Lappe et al., 2010). Along similar lines, we recently proposed that compression may reflect an underestimation of distance due to a lack of perceived apparent motion seen between the reference and the subsequently presented probe (Zimmermann et al., 2014a; Born et al., 2015). If detected, the perception of motion may help determine the spatial separation between the two bars as by definition, motion means covering a distance in a certain time. Without perceived apparent motion, another vital distance cue is missing. Further, apparent motion, just as compression, is modulated by luminance contrast (Anstis and Mather, 1985).

### Transient Disruptions of the Visual Input Stream

In previous work (Zimmermann et al., 2014a; Born et al., 2015), we proposed that compression reflects a process that matches corresponding objects across space and time (Ullman, 1979). After disturbances in the visual input stream, the visual system needs to retrieve the new locations of current targets to connect locations and identities before and after, thus bridging the interruption. In a similar vein, models of saccadic compression critically assume a role for the integration of pre- with post-saccadic visual input (Lappe et al., 2010; Cicchini et al., 2013). Other phenomena, such as change blindness (Rensink et al., 1997) demonstrate the challenges of such visual disruptions for perception. The current results suggest that even the onset of a weak signal on its own is sufficient to obtain compression effects. Somehow the strong reference signal is linked to the weak probe and biases its location. The arrival of the probe signal requires a verification to see if it is additional information about the existing reference or a new object. Possibly the probe signal is erroneously taken as new evidence about the location of the reference and their locations interact based on this tentative correspondence. However, the location interaction is not critically dependent on an additional transient from a mask or a saccade. Evidence for the role of correspondence matching comes from studies demonstrating that the strength as well as the direction of compression effects depends on the similarity between the available visual references and the probe (Cicchini et al., 2013; Zimmermann et al., 2014a).

### Neurophysiology and Computational Models

As mentioned in the introduction, many researchers have suggested that spatial compression reflects interactions between extraretinal oculomotor signals and visual input. Initially, most models were inspired by neurophysiological findings of cells in the lateral intraparietal area that shifted their sensitivity in saccade direction just prior to the eye movement (Duhamel et al., 1992). More recently, systematic receptive field shifts observed in frontal eye field and V4 neurons not parallel to the saccade, but biased from all directions towards the saccade target (Tolias et al., 2001; Zirnsak et al., 2014) have been considered (Hamker et al., 2008; Zirnsak and Moore, 2014). There is to date no undisputed framework to explain how transient receptive field changes mediate the perceptual effects. Interestingly, most models focus on explaining why compression is centered on the saccade target. It is possible that instead, a more important implication of the observed neurophysiological phenomena prior to saccades is that they reduce the quality of encoding of the probe’s spatial signal (see also Gottlieb et al., 1998).

## Conclusion

In sum, we observed a strong dependency of compression on probe visibility in the context of saccades and masks. Although saccades and masks produced compression even at close to perfect probe detection rates, low visibility levels of the probe were sufficient to induce compression-like mislocalizations even in the absence of such visual disruptions. We conclude that the behavioral phenomenon of compression of space is not closely linked to mechanisms mediating spatial updating of retinotopic maps across saccades. Rather, compression may reflect how the visual system localizes weak targets in the context of highly visible stimuli.

## Author Contributions

SB and HMK designed, performed experiments, analyzed data and prepared figures; SB, HMK, EZ and PC interpreted results of experiments; SB drafted manuscript; SB, HMK, EZ and PC edited, revised manuscript and approved final version of manuscript.

## Conflict of Interest Statement

The authors declare that the research was conducted in the absence of any commercial or financial relationships that could be construed as a potential conflict of interest.
